# Nourishing neonatal piglets with synthetic milk and *Lactobacillus* sp. at birth highly modifies the gut microbial communities at the post-weaning stage

**DOI:** 10.3389/fmicb.2022.1044256

**Published:** 2022-11-30

**Authors:** Vetriselvi Sampath, Jun Ho Song, Jinuk Jeong, Seyoung Mun, Kyudong Han, In Ho Kim

**Affiliations:** ^1^Department of Animal Resource and Science, Dankook University, Cheonan, South Korea; ^2^Department of Bio-Convergence Engineering, Dankook University, Cheonan, South Korea; ^3^Department of Microbiology, College of Science and Technology, Dankook University, Cheonan, South Korea; ^4^Center for Bio Medical Engineering Core Facility, Dankook University, Cheonan, South Korea

**Keywords:** growth performance, gut microbiome, piglets, probiotics, synthetic milk

## Abstract

The importance of probiotics in pig production is widely recognized. However, the precise role of probiotics in regulating the gut microbiota of piglets has not been assessed extensively. Therefore, we intend to examine whether suckling pigs ingesting with synthetic milk (SM) and probiotics along with mother milk has a carryover effect on its growth and gut health at the post-weaning stage. A total of 40 [Duroc× (Yorkshire× Landrace)] neonates with an initial BW of 1.49 ± 0.28 kg were assigned to one of two treatments groups: control (CON) and treatment (TRT). Control group piglets were nourished with synthetic milk, while TRT group piglets were nourished SM with (1 × 10^9^ CFU/g) *Lactobacillus* sp. probiotics. The treatment group piglets showed higher (*p* < 0.05) body weight and daily gain at week 3 than the CON group piglets. 16S metagenome sequencing showed average demultiplexed reads and denoised reads counts of 157,399 and 74,945, respectively. The total ASV taxonomy number classified with a confidence threshold > 70% (default) on sequence alignment with the SILVA v138 reference database was 4,474. During week 1, *Escherichia-Shigella, Clostridium sensu stricto 1*, and *Bacteroides* were confirmed as the major dominant bacterial genera in both the groups at the genus level. However, during week 2, the relative proportion of *Escherichia-Shigella, Clostridium sensu stricto 1*, and *Proteobacteria* was decreased, while that of *Lactobacillus* and *Bacteroidota* was increased in pigs receiving the probiotic supplement. During weeks 2 and 3, *Firmicutes, Proteobacteria*, and *Bacteroidota* phyla were dominant in both groups. During week 6, the relative proportion of *Proteobacteria* was slightly increased in both groups. Furthermore, *Prevotella* was confirmed as the major dominant bacterial genus in both groups during weeks 3 and 6. This study suggests that nourishing neonatal piglets with synthetic milk and *Lactobacillus* sp. probiotics from birth to 21 days would be beneficial to enhance the gut health of piglets and to overcome post-weaning mortality.

## Introduction

Early weaning is a viable way to achieve better breeding and economic benefits in modern intensive swine production (Patil et al., 2015). However, there are growing health and welfare consequences due to large litter sizes, and the optimal management of large litters turns out to be a challenging task for pig producers (Pustal et al., 2015). Especially, the first 24 h after birth is a crucial period as piglets are born with low body energy stores and/or without immunoglobulins, and it takes at least 2 weeks for piglets to attain active immunity. The only way to protect them in these life-threatening weeks is through the first milk comsumption (colostrum). Over the past decades, different fostering practices were followed by farm owners to improve survivability and reduce the mortality of piglets, among which cross-fostering (within 12 h) has become a common method. Although cross-fostering is considered an effective strategy, the total number of accessible teats on newly farrowed sows in a prolific herd may not be sufficient for the number of newborns, and thus, some piglets face severe starvation during lactation, which ultimately results in poor growth performance and eventually leads to death (Rutherford et al., 2013). This circumstance has prompted scientists, farm owners, and pig industrialists to find a suitable complement to colostrum that could provide additional nutritional interventions to improve the growth performance of piglets. Lehrer et al. (1949) proposed the provision of synthetic milk (SM) as an effective solution for improving the growth capacity of neonates. Following this study, Ruurd et al. (1996) reported that feeding milk replacers from day 14–28 increased the daily weight gain of pigs at weaning. In addition, Novotni-Dankó et al. (2015) and De Greeff et al. (2016) stated that the administration of SM increased body weight and reduced pre-weaning mortality in piglets, respectively. Concurrently, a nutritional approach to the use of probiotics has gained more attention since the 1970's (Fuller, 1977), and this finding was corroborated by many researchers (Nguyen et al., 2019; Sampath et al., 2022) who suggested that a probiotic live microorganism used as a therapeutic adjuvant could improve the feeding behavior and reduce the morbidity and mortality of animals. Such probiotics were recently used in different strains with different efficacies; some of them were established to provide certain benefits to the host (Patil et al., 2015). One of the most widely used probiotic strains is lactic and acetic acid bacteria, which has been shown to produce antimicrobial substances against homologous strains and to produce microbicidal substances against gastric and intestinal pathogens (Ljungh and Wadström, 2006). Notably, *Lactobacillus* strains are highly effective in diminishing *Escherichia coli, Salmonella*, and coliform counts in poultry (Ramasamy et al., 2009; Hardy et al., 2013) and *Clostridium* sp. in piglets (Liu et al., 2014). Nguyen et al. (2019) stated that weaning pigs that were fed a probiotic mixture supplement had better growth performance and nutrient digestibility. Similarly, Jäger et al. (2018) observed that feeding a diet supplemented with *Bacillus* culture to pigs enhanced nutrient absorption, by improving gut health.

Gut microbiome plays an important role in improving the health of the host (Milani et al., 2017). The gastrointestinal tract (GIT) of neonatal animals is free from “germs” at birth (Benson et al., 2010); however, the GIT of newborns can be easily inhabited by the microbial communities either from the dam or from the environment. The microbial population existing within the GIT of animals is diverse (Yeoman and White, 2014), and these bacteria mainly belong to bacterial phyla like Firmicutes and Bacteroidetes. Notably, the phylum Firmicutes comprises genera including *Bacillus, Lactobacillus, Enterococcus, Clostridium*, and *Ruminococcus*, while the phylum Bacteroidetes consists of predominant genera like *Bacteroides* and *Prevotella* (Rinninella et al., 2019). Although the phylum *Actinobacteria* is comparably less abundant, it is mainly characterized by the genus *Bifidobacterium* (Arumugam et al., 2011). *Firmicutes* are largely found in the hindgut of pigs, while *Bacteroidetes* are found in the cecum of chickens. In general, these two phyla are dominant in the healthy host, whereas the lower ratio of Firmicutes to Bacteroidetes may reflect a reduction in the microbial diversity in the GIT (Ling et al., 2014). So far, several studies have explored the potential role of probiotics in animals and have demonstrated that feed additive probiotics increase the abundance and colonization of beneficial bacteria, by improving the gut health of the host. For instance, Lu et al. (2018) reported that the inclusion of a probiotic complex (*Enterococcus faecium* DSM 7134, *Bacillus subtilis* AS1.836, and *Lactobacillus paracasei* L9) significantly increased the relative abundances of *Prevotella_1* and *Lactobacillus* and reduced the relative abundances of *Bacteroidales* and *Clostridium_sensu_1* in weaning pigs. Moreover, Sugiharto et al. (2015) stated that SM modulates the mucosal immunology and the abundance of microbiota in neonatal piglets and reduces the abundance of *Escherichia* and diarrhea frequency in piglets after weaning. The aforementioned studies prompted us to hypothesize that nourishing neonatal piglets with SM and a probiotic supplement at their early days might improve their growth and intestinal health at the post-weaning stage. Although the administration of SM and a probiotic supplement showed a positive result in different studies on pigs of different age categories, to the best of our knowledge, this study would be the first to explore the nutritional effect of the supplementation of SM with a probiotic in the neonate diet. Therefore, to test this hypothesis, we aimed to assess the effects of supplementation of synthetic milk with a probiotic on the growth performance and gut health of pigs through the determination of body weight and fecal microbiota composition.

## Materials and methods

### Ethical approval

Husbandry practices strictly conformed to the guidelines of animal welfare, and the experimental protocol (No: Dk-2-2029) was approved by the Animal Care and Use Committee of Dankook University (Cheonan, South Korea) prior to the trial.

### Source of probiotics

The probiotic (*Lactobacillus* sp.) additive used in this study was procured from ShinGuen-Bio Co. Ltd (South Korea). Before fermenting the enzyme solution, the probiotic was cultured at a temperature of 30°C under anaerobic conditions in a customized medium containing protease, yeast extract, etc. After the initial culture, *Lactobacillus* sp. was fermented for 6 months, during which all molasses were decomposed at 30°C using sterilized molasses as a food source. To obtain essential enzymes for feed digestion and decomposition, a secondary fermentation process was carried out, which included freeze-drying of *Lactobacillus* sp. powder (1 × 10^9^ colony-forming units (CFU)/g). Following the secondary fermentation process, the contents of essential enzymes such as acid protease (100 U/g), neutral protease (40 U/g), cellulase (10 U/g), and lipase (8 U/g) were measured to identify the components of the enzyme, which was performed at the Institute of Agriculture Science (Chungnam National University, Republic of Korea).

### Animals, experimental design, diets, and feeding schedule

This experiment was carried out at “Dankook University- sow experimental farm”, Gonju. A total of four healthy sows were artificially inseminated (AI) two times (12–24 h) with the semen of Duroc boars. On day 105 of pregnancy, sows were weighed individually, moved to farrowing crates (113 × 62 × 168 cm) equipped with heating beds for the piglets, and kept there until the end of the lactation period. From day 107 of gestation to farrowing, the sows were fed soybean meal-based basal (2.5 kg/ d) diets; they were not fed on the farrowing day; and after the farrowing day to weaning, the sow were fed with lactation diet. All the diets were formulated to meet the nutrient requirements recommended by the National Research Council [Nutrient research council (NRC), 2012]. Shortly after parturition, regular husbandry practices were instituted. Within 12 h, the piglets were cross-fostered, and for 36 h, the neonates consumed only sow colostrum. The breeding room temperature was maintained at 27°C.

On day 3 post-partum, a total of 40 healthy crossbred [Duroc× (Yorkshire× Landrace)] neonates along with their dam were assigned to one of the two dietary treatment groups (20 piglets/treatment, 10 piglets/pen), that is, control (CON) and treatment (TRT). The average initial body weights (1.49 ± 0.28 kg) of these two groups were similar. Until week 3, the neonates in the CON group were allowed to consume synthetic milk (SM), while the TRT group piglets were fed SM with (1 × 10^9^ CFU/g) *Lactobacillus* sp. supplement. Both the groups had *ad libitum* access to mother milk until day 21. The composition of SM is presented in Table 1. The milk replacer was prepared by mixing 100 g of SM powder with 1 L of warm water (approximately 30 °C), and the probiotic supplement was added to it and the mixture was fed to piglets every 3 h in a bowl.

**Table 1:** Composition of the synthetic milk.

**Ingredients (%)**	**Suckling pigs**
Isolated soy protein	9.20
Milk replacer	37.50
Whey powder	39.59
Lactose	6.40
Lysine 78%	0.57
Methionine 99%	0.36
Threonine 98%	0.26
Tryptophan 10%	0.90
Sugar	3.50
Glucose	1.00
Sweetener	0.02
Mineral^a^ and vitamin^b^ premix	0.20
Organic acid	0.50
Total	100.00
**Analyzed values** (%)
Moisture	3.95
Crude protein	20.56
Crude fat	6.03
Crud fiber	0.04
Crude ash	6.46
Calcium	0.69
Phosphorus	0.59
Digestible energy	3,948
Lysine	1.88
Methionine	0.63
Threonine	1.28
Tryptophan	0.34
Vitamin A	30,375
Lactose	50.67

a Fe, 50 mg as ferrous sulfate; Cu, 8.5 mg as copper sulfate; Mn, 8.5 mg as manganese oxide; Zn, 50 mg as zinc oxide; I, 0.25 mg as potassium iodide; and Se, 0.15 mg as sodium selenite.

b Vitamin D3, 2,000 IU; vitamin E, 20 IU; vitamin K3, 2 mg; vitamin B1, 3 mg; vitamin B2, 6 mg; vitamin B6, 3 mg; vitamin B12, 0.025 mg; biotin, 0.1 mg; folic acid, 1 mg; niacin, 25 mg; D-calcium pantothenate, 12.5 mg.

### Sampling and clinical analysis

#### Growth performance

For 3 weeks, the milk supplements were fed to the piglets. The individual body weight (BW) of piglets was measured at birth, end of week 1, and week 3. The milk consumed and the leftover in the bowl were measured and recorded on a pen basis to calculate the average daily feed intake (ADFI) and average daily gain (ADG). At the time of weaning (day 21), the 40 piglets were separated from their mothers, housed in a weaning facility (20 piglets/treatment with five replicates and four pigs/pen), and fed a commercial maize–soy bean meal-based diet (Table 2) until week 6 [Nutrient research council (NRC), 2012].

**Table 2:** Composition of weaning piglet diets (as-fed basis).

**Ingredients (%)**
Corn	39.22
Soybean meal	16.42
Fermented soybean meal	5.00
Spray dried plasma protein (SDPP)	6.00
Tallow	2.49
lactose	13.46
Sugar	3.00
Whey protein	11.00
Monocalcium phosphate	0.90
Limestone	1.17
Salt	0.20
Methionine (99%)	0.22
Lysine	0.49
Mineral mix^a^	0.20
Vitamin mix^b^	0.20
Choline (25%)	0.03
Total	100.00
Analyzed value	
Crude protein, %	20.00
Calcium, %	0.80
Phosphorus, %	0.60
Lysine, %	1.60
Methionine, %	0.48
Metabolizable energy (ME), kcal/kg	3,450
FAT, %	4.18
Lactose, %	20.00

a Provided per kg diet: Fe, 100 mg as ferrous sulfate; Cu, 17 mg as copper sulfate; Mn, 17 mg as manganese oxide; Zn, 100 mg as zinc oxide; I, 0.5 mg as potassium iodide; and Se, 0.3 mg as sodium selenite.

b Provided per kg diet: vitamin A, 10,800 IU; vitamin D3, 4,000 IU; vitamin E, 40 IU; vitamin K3, 4 mg; vitamin B1, 6 mg; vitamin B2, 12 mg; vitamin B6, 6 mg; vitamin B12, 0.05 mg; biotin, 0.2 mg; folic acid, 2 mg; niacin, 50 mg; D-calcium pantothenate, 25 mg.

#### Metagenomic DNA extraction and 16S RRNA sequencing of fecal samples

At the end of weeks 1, 2, 3, and 6, 200 g (each treatment) of fresh fecal specimens were randomly collected from 20 healthy piglets (10 pigs/group) by rectal palpation, placed in a sterile tube, and taken to the laboratory. Metagenomic DNA (mDNA) was extracted according to the manufacturer's instructions with some modification. A measure of 100 mg of the fecal samples was mixed with 1.4 ml of lysis buffer in a 2-ml tube and vortexed until the samples were thoroughly homogenized. The samples were subsequently mixed with 0.2 g of sterile zirconia/silica beads. Next, the samples were processed on a TissueLyser for 6 min at 30 Hz. Lysis was carried out at a temperature of 95°C for 5 min. Finally, DNA was extracted using the QIAamp Power Fecal Kit (Qiagen, Germany) following the manufacturer's instructions and eluted using 100 μl of elution buffer provided in the kit. The following protocol describes the steps carried out to amplify the targeted 16S rRNA gene V3-V4 regions of the bacteria present in each of the collected samples, as well as processes required to prepare the purified DNA fragments for next-generation sequencing: Each sequenced sample was prepared according to the Illumina 16s amplicon sequencing library protocols. The quantification and qualification of the DNA samples were measured using a Qubit fluorometer and Nanodrop equipment, respectively. The preparation of a library of amplicons consisting of 16S rRNA gene and sequencing was carried out on the Illumina MiSeq platform. The first 16S amplicon primer sequences were as follows: the 16S V3-V4 forward primer was 5′ - TCGTCGGCAGCGTCAGATGTGTATAAGAGACAG−3′; the 16S V3-V4 reverse primer was 5′ - GTCTCGTGGGCTCGGAGATGTGTATAAGAGACAG−3′. Input gDNA (10 ng) was amplified with 16S V3-V4 primers, and second limited-cycle amplification was performed to add multiplexing indices (barcode) and Illumina sequencing adapters. The final products were normalized and pooled using the Qubit fluorometer, and the library sizes were verified using a TapeStation system (Agilent, CA, USA). Finally, high-throughput amplicon sequencing was conducted on the MiSeq™ platform (Illumina, San Diego, CA, USA).

#### Microbial 16S rRNA gene sequencing data analysis

To normalize the amplicon sequence variant (ASV) matrix to ensure that microbial reads produced by high-throughput sequencing have the same total number of reads in all samples, we performed rarefaction curves to determine if all existing ASVs were recovered sufficiently. Microbial 16S rRNA sequencing data were analyzed on the QIIME 2™ next-generation microbiome bioinformatics platform (Bolyen et al., 2019). All QIIME2 input data were in the form of QIIME2 artifacts, which contain information about the data types and sources. All Fastq reads were imported using the “tools imports” command. Sequence quality control and feature table construction were completed using the Divisive Amplicon Denoising Algorithm 2 (DADA2) QIIME 2 plugin, which detects and corrects amplicon errors and filters out PhiX chimeric sequences. Sequences containing ambiguous base calls and sequences <100 bp were trimmed to minimize the effects of random sequencing errors. After the denoising step, the feature data were specified *via* the pre-trained naive Bayes classifier artifact using the machine learning Python library scikit-learn in the QIIME2 pipeline. This classifier artifact was trained against SILVA database v138 trimmed to contain only the V3-V4 hypervariable regions and pre-clustered at a 99% sequence identity, and then a 70% (default) sequence identity was utilized as a confidence threshold for taxonomic classification (Quast et al., 2012).

### Statistical analysis

Experimental data were analyzed by using SAS 9.2 software (SAS Inst. Inc., Cary, NC, USA) Before the analysis, all data were tested for normality, and growth performance (until day 21) was analyzed using the Shapiro–Wilk test with litters (ADG and ADFI) and individual piglets (BW, *n* = 20/ treatment) as the experimental unit. From weeks 4 to 6, data were analyzed through a completely randomized design using the pens as an experimental unit. The statistical trend was 0.05 *P* < 0.1 and ^*^P < 0.05. Fecal sample diversity analyses were performed using the “diversity” QIIME2 plugin to determine alpha- and beta-diversities. Alpha-diversity was measured by observing the ASVs, Chao1 index, Shannon index, and Simpson index, and Pielou_evenness indices, which account for richness and evenness. Beta-diversity was measured using principal coordinate analysis of both unweighted UniFrac and Bray–Curtis distances. The Mann–Whitney statistical test was used to compare the microbial communities to identify significant differences. The Mann–Whitney statistical significance was used to evaluate the statistical similarity between the two methods and the asterisk (^*^) mark was used to denote if the P-value was < 0.05. This non-parametric statistical analysis was performed by using GraphPad PRISM v8 (GraphPad Software Inc., CA, USA).

## Result and discussion

### Growth performance

**Figure 1:**
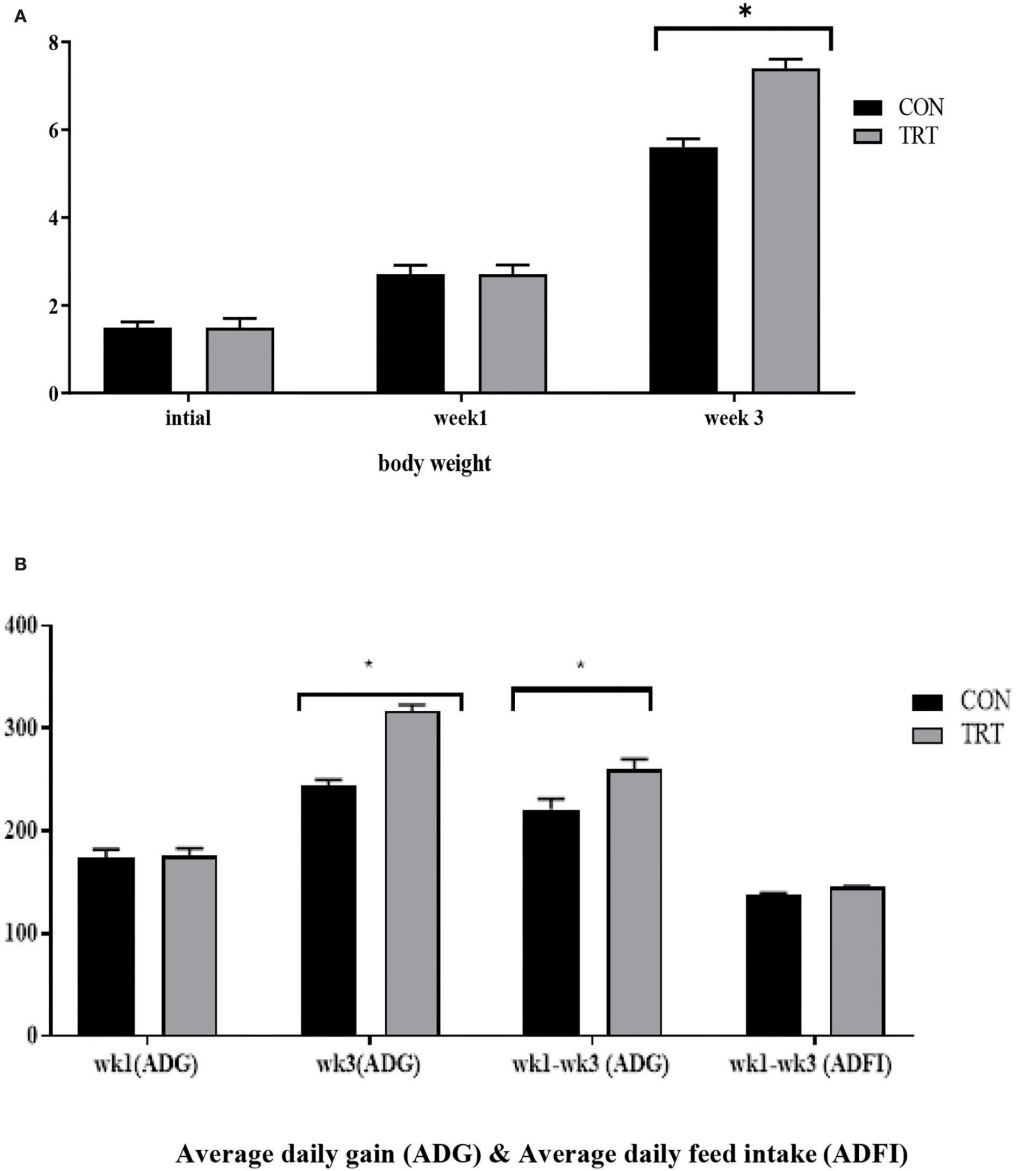
**(A,B)** Increase in body weight (BW, kg) and ADG (g) of piglets following administration of probiotic supplements. The x- and y-axes **(B)** indicate the ADG scale, defined as the average weight a market animal will gain each day during the feeding period, and the experimental participant ID, respectively. The black and gray colors denote control and treatment groups, respectively. *Denotes statistically significant *p* < 0.05.

During the suckling period, neonates can only digest sow milk and fully utilize its nutrients but cannot utilize the full nutritional benefits from creep feed because of their immature digestive systems. Previously, several researchers have reported that dietary changes during early weaning alter the intestinal microbiota composition of suckling pigs (Zheng et al., 2021). For instance, Ruurd et al. (1996) reported that feeding milk replacers from days 14 to 28 increased the daily weight gain of pigs at weaning. Similarly, Sugiharto et al. (2015) demonstrated that the provision of SM enhanced the gut microbial population in suckling piglets. Furthermore, Liao and Nyachoti (2017) stated that the inclusion of probiotics in the diet enhanced the abundance of beneficial bacteria, achieving optimal nutrient degradation and a healthy intestinal tract. Comparable findings were observed in the present study. The body weight (BW) of the piglets was not influenced by the experimental diet at birth or at week 1, whereas during week 3, the BW of the treatment group piglets significantly increased with probiotic supplementation. Moreover, the piglets that received probiotic supplements showed higher daily gains at week 3 and during the overall experimental period (Figures 1A,B), and this result was consistent with Gebert et al. (2011), who replaced the milk supplement with a *Lactobacillus* probiotic and obtained a positive effect on pre-weaning animals. In an earlier study, Kenny et al. (2011) reported that the provision of probiotics from birth could potentially establish a life-long health benefit in newborn piglets. While Nowland et al. (2019) stated that intrauterine microbiota colonization or fetal exposure to the maternal gut microbiota significantly affects the development of intestinal functions and the immune system by altering post-partum colonization. Thus, the probable reason for enhanced growth performance at early weaning might be due to an appetizing diet that is enriched with 37.50% synthetic milk and 39.59% whey protein. As whey protein contains a high amount of α-lactalbumin and β-lactoglobulin (Pescuma et al., 2008), we thought that it might help the host to improve its performance by modulating its intestinal microbiota composition. To date, several academics have tried to find a proper solution to overcome post-weaning stress in piglets, which could cause huge economic losses to the swine industry (Liao et al., 2020). Shi et al. (2018) reported that formula milk supplementation improved the growth performance of piglets by reducing the incidence of diarrhea pre- and post-weaning, and this outcome proved that gut microbiota plays an important role in these changes. In the current study, the administration of SM with a probiotic increased the average daily weight gain of the piglets during the pre-weaning period. In addition, the relative proportion of *Lactobacillus* was enriched in the piglets receiving probiotic supplementation, and this finding greatly supports the achievement of better growth performance and the improved gut health of piglets by reducing pathogenic bacterial inhibition. Baktavachalam et al. (2015) reported that milk substitutions improved the feed intake of piglets. However, in this study, no difference was observed in the average daily feed intake, and this lack of ADFI become the main reason for no significant improvement in the BW of piglets at the post-weaning stage. Another reason for no improvement in the daily feed intake of piglets from birth to day 21 (week 3) might be social factors or their suckling behavior. In addition, no differences were observed in growth parameters (from weeks 4 to 6; results not included), while differences in microbiota or the dominance of a bacterial genus were observed during week 6. Early weaning piglets, particularly 3- to 4-week-old piglets, are subjected to numerous stressors, like separation from their dam, co-mingling with new litter mates, and a change of diet from liquid to solid. Several studies (McGone et al., 1987; Rundgren and Löfquist, 1989) have reported that this circumstance led piglets to suffer from lower appetite, body weight loss, improper digestive function, and post-weaning diarrhea. As this is a preliminary study, we speculate that the lack of growth performance after day 21 may be attributed to any of these stress factors, and the exact cause for this outcome is currently unknown; therefore, further research on growth performance parameters will be performed with a high dose of the additive.

### 16S metagenome sequencing

Recently, the interaction between the gut microbiome and piglet health has attracted research interest from many scientists (Kim and Duarte, 2021). Also, robust microbial communities at the early stage of piglets are crucial for developing gut functions and the immune system (Chen et al., 2018). To compare the differences in bacterial composition between two piglet groups (CON and TRT), we successfully prepared metagenome sequencing libraries by targeting the bacterial 16S V3-V4 region. Average demultiplexed reads, and denoised read counts were 157,399 and 74,945, respectively (Supplementary Table S1). The total ASV taxonomy number classified with a confidence threshold > 70% (default) for sequence alignment with the SILVA v138 reference database was 4,474 (Supplementary Table S2).

### Comparison of microbial diversity (Alpha and Beta) between two treatment groups

**Figure 2:**
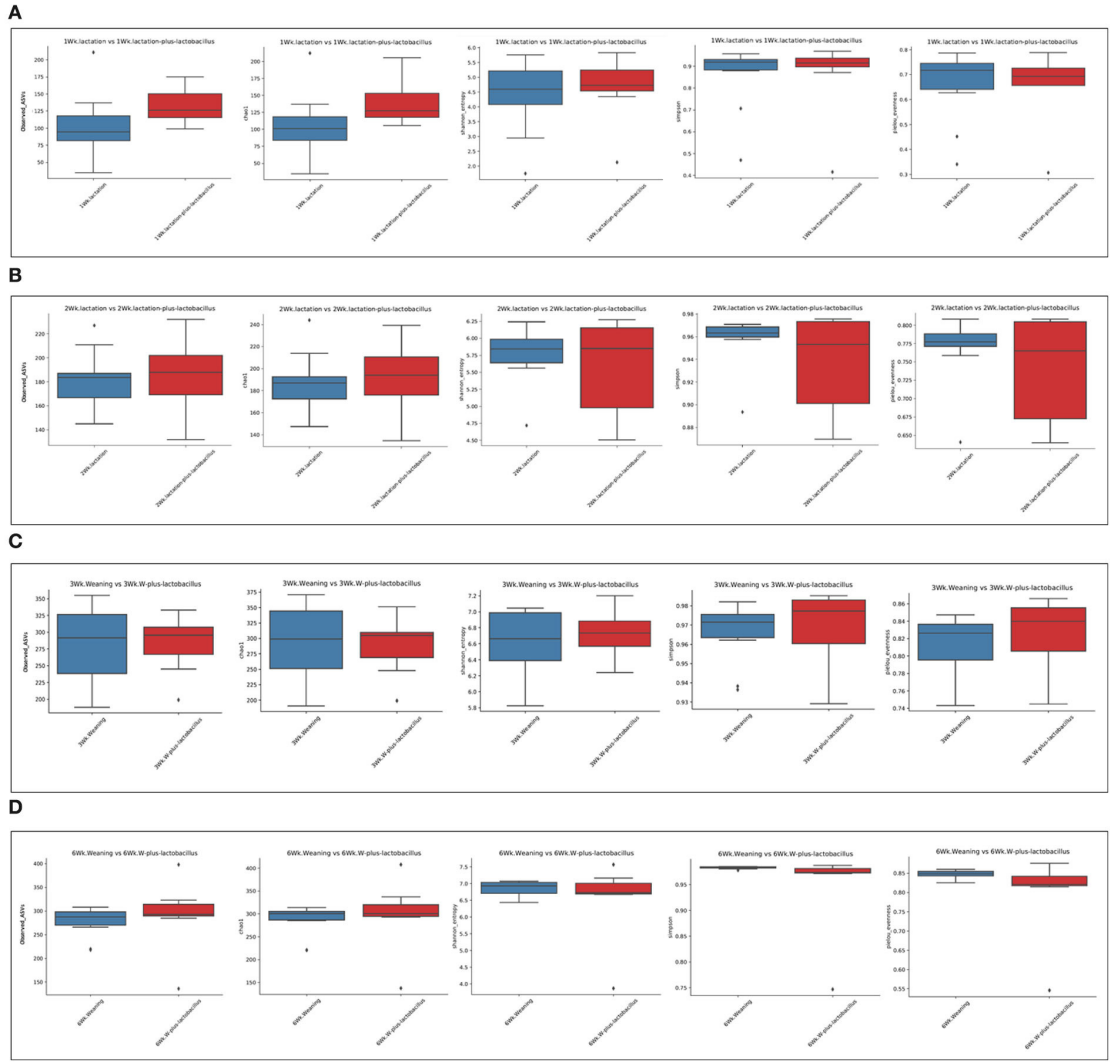
Alpha-diversity analysis of control and treatment gut microbiome. These box plots show the alpha-diversity estimation scores for each period [**(A)** week 1, **(B)** week 2, **(C)** week 3, and **(D)** week 6]. Each alpha-diversity was calculated using 1: Observed_ASVs, 2: Chao 1, 3: Shannon_entrophy, 4: Simpson, and 5: Pielou_evenness indices in order.

**Figure 3:**
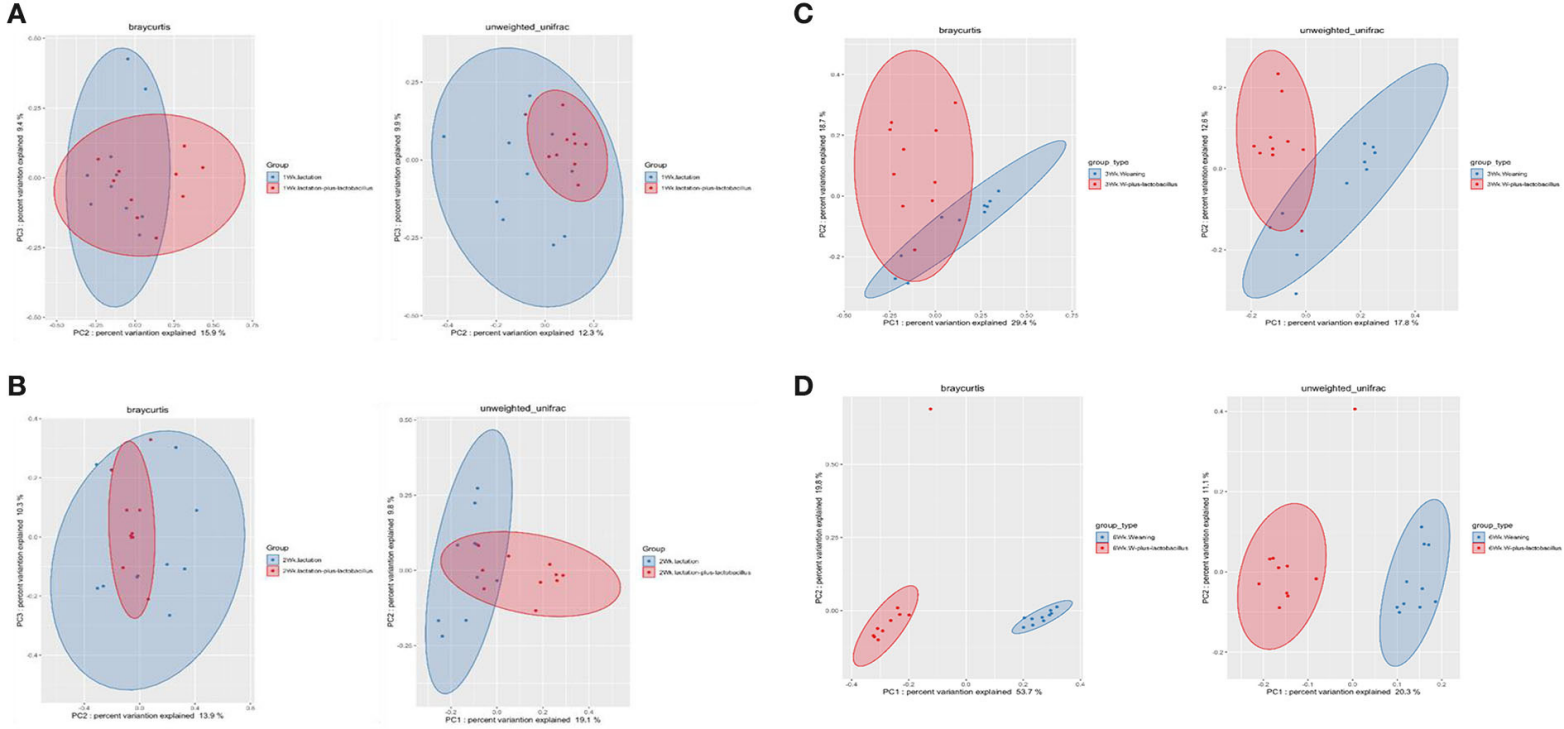
Beta-diversity analysis of four dependent periods between each piglet. Microbial beta-diversity analysis measured by both Bray–Curtis distance and unweighted UniFrac distance matrix for all samples. Time-series comparison analysis between control and treatment groups at **(A)** 1, **(B)** 2, **(C)** 3, and **(D)** 6 weeks are represented. The circular clusters represent the distance between control (blue) and treatment (red) groups based on the microbial diversity between the groups and the similarity of the gut microbiomes.

To compare the richness and evenness of the intestinal microbiota between the two piglet groups, we estimated the alpha-diversity by using Observed_OTUs, Chao1, Shannon, Simpson, and Pielou_e alpha-diversity indices (Table 3). In these comparison results for each growth week, we observed that microbial richness was higher in the TRT group at weeks 1 and 2 (as evidenced by the Observed_ASVs and Chao1 indices). In contrast, a difference in the alpha-diversity of comparison groups for other weeks was not identified (Figure 2). In addition, the diversity score in the control group at week 6 was high in some alpha-diversity indices (the Simpson and Pielou_e indices); this was due to the lower estimated diversity score of minority samples in the TRT group than in the average value (Table 3). We confirmed these alpha-diversity comparisons by the results from the fact that nourishing piglets with *Lactobacillus* probiotics may parallelly be affected by an increase in the diversity of early intestinal microbial species in the piglets. Next, we clustered the estimated microbial compositions for each growth week between the two comparison groups through beta-diversity analysis. As a result, we confirmed that the microbial compositions of each sample were clearly divided between the lactation and weaning intake periods. In addition, it has been confirmed that microbial cluster changes in the inter-group were evident, depending on whether the probiotic product was taken or not (Figure 3).

**Table 3:** Alpha-diversity statistics using the Mann–Whitney test for each comparison group.

**Alpha-diversity index**	**1Wk_Lactation**	**1Wk_Lactation-plus-*Lactobacillus***	**Mann-Whitney; p**	**Statistical significant**
Observed_ASVs	104.4 ± 46.33	133.2 ± 25.78	0.0171	*
Chao1	106.4 ± 46.09	138.96 ± 31.93	0.0156	*
Shannon	4.38 ± 1.23	4.68 ± 1.01	0.2363	ns
Simpson	0.86 ± 0.15	0.87 ± 0.16	0.4549	ns
pielou_evenness	0.66 ± 0.15	0.66 ± 0.13	0.3957	ns
**Alpha-diversity index**	**2Wk_Lactation**	**2Wk_Lactation-plus-** ***Lactobacillus***	**Mann-Whitney; p**	**Statistical significant**
Observed_ASVs	180.6 ± 25.55	185.0 ± 29.2	0.2849	ns
Chao1	185.83 ± 28.77	191.12 ± 31.19	0.2854	ns
Shannon	5.75 ± 0.42	5.58 ± 0.67	0.4549	ns
Simpson	0.96 ± 0.02	0.94 ± 0.04	0.4549	ns
pielou_evenness	0.77 ± 0.05	0.74 ± 0.07	0.4549	ns
**Alpha-diversity index**	**3Wk_Weaning**	**3Wk_Weaning-plus-** ***Lactobacillus***	**Mann-Whitney; p**	**Statistical significant**
Observed_ASVs	282 ± 55.67	286 ± 41.19	0.4849	ns
Chao1	293.46 ± 60.4	291.42 ± 44.54	0.4251	ns
Shannon	6.59 ± 0.44	6.73 ± 0.3	0.3388	ns
Simpson	0.97 ± 0.02	0.97 ± 0.02	0.2137	ns
pielou_evenness	0.81 ± 0.03	0.83 ± 0.04	0.0929	ns
**Alpha-diversity index**	**6Wk_Weaning**	**6Wk_Weaning-plus-** ***Lactobacillus***	**Mann-Whitney; p**	**Statistically significant**
Observed_ASVs	276.5 ± 32.54	293.4 ± 64.5	0.0864	ns
Chao1	285.42 ± 35.07	300.31 ± 66.86	0.1724	ns
Shannon	6.86 ± 0.22	6.61 ± 1.0	0.4251	ns
Simpson	0.98 ± 0.0	0.95 ± 0.07	0.0226	*
pielou_evenness	0.85 ± 0.01	0.81 ± 0.09	0.0320	*

The statistical similarity between two methods is denoted by an asterisk (*) mark.

### Determining dramatic changes in the gut microbiome of piglets using probiotics supplements

Intestines of piglets are highly colonized by a complex community of microorganisms composed mainly of bacteria (Isaacson and Kim, 2012). Predominantly, the early postnatal period is thought to be a “critical window” for modifying the gut microbiota, as it is the period in which the microbiome is more responsive to internal and external stimuli (AI-Shawi et al., 2020). In this study, we performed a relative abundance analysis to confirm the difference in the proportion of intestinal bacterial strains between the two comparative groups of each growth week (Supplementary Table S3). The intestinal bacterial community of the piglets in each growth week was classified into taxonomic ranks based on the SILVA v138 16S rRNA gene database. *Firmicutes* and *Bacteroidetes* were the most dominant phyla of bacteria in the fecal samples of the piglets (Lu et al., 2018). Thus, microbiome composition in the fecal sample was explored, and a substantial difference was observed between the composition in genus and phylum levels. The relative proportions of *Firmicutes, Proteobacteria*, and *Bacteroidota* phyla were dominant in both comparison groups during the first and second growth weeks from birth. This result was projected because of the colon; as they are in a strictly anaerobic environment, most of the species within these phyla are anaerobic (Kim and Isaacson, 2015). In particular, from the first to third growth week, the piglets receiving SM with *lactobacillus* strains exhibited an increased relative proportion of *Bacteroidota*, whereas that of *Proteobacteria* decreased. However, a slight increase in the relative proportion of *Proteobacteria* was observed in the TRT group at the end of week 6. Considering that an adequate balance between *Bacteroidota* and *Firmicutes* phyla is an evaluation point for the intestinal bacterial composition in healthy animals, we could confirm that probiotics positively affected the piglet intestinal microbial environment in the early growth period (Stojanov et al., 2020). Duarte and Kim (2022) reported that piglets with a higher body weight had a greater abundance of *Bacteroidetes, Bacteroides*, and *Ruminococcaceae* and lower proportions of *Actinobacillus porcinus* and *Lactobacillus amylovorus* than piglets with low body weights (Supplementary Figure S1). Luo et al. (2022) reported that changes in the gut microbial community were highly correlated with basal diet composition. Especially, a diet rich in fat and protein was capable of increasing the ratio of *Firmicutes* to *Bacteroides* in the gut of animals and humans (Magne et al., 2020). From this, we speculate that the increased abundance of *Firmicutes* and *Bacteroidota* at the phylum level is mainly attributed to the addition of milk replacers, which contained 20.56% protein and 6.03% fat.

In this study, *Escherichia*-*Shigella, Clostridium sensu stricto* 1, and *Bacteroides* were confirmed as the major dominant bacterial genera at the genus level in both the CON and TRT groups during the first growth week. Moreover, during the second growth week, the relative proportions of *Escherichia*-*Shigella* and *Clostridium sensu stricto* 1 decreased, whereas that of *Lactobacillus* increased. Based on the recent finding that epithelial inflammation in piglets correlates with the proportion of *Clostridium sensu stricto* 1 in the intestinal mucosa, we noted that the relative proportion of *Clostridium sensu stricto* 1 decreased in both groups during these two growth periods, which agrees with Wang et al. (2018). *Prevotella* was confirmed as the major dominant bacterial genus in both comparison groups at the third and sixth growth weeks. The proposed reason for this variation could be changes in the staple weaning diet to include fiber content (Kovatcheva-Datchary et al., 2015). However, no difference was found in the relative bacterial proportion between the two comparison groups in the third growth week, or in the sixth week, while *Clostridium sensor stricto 1* increased in the TRT group pigs (Supplementary Figure S2).

**Figure 4:**
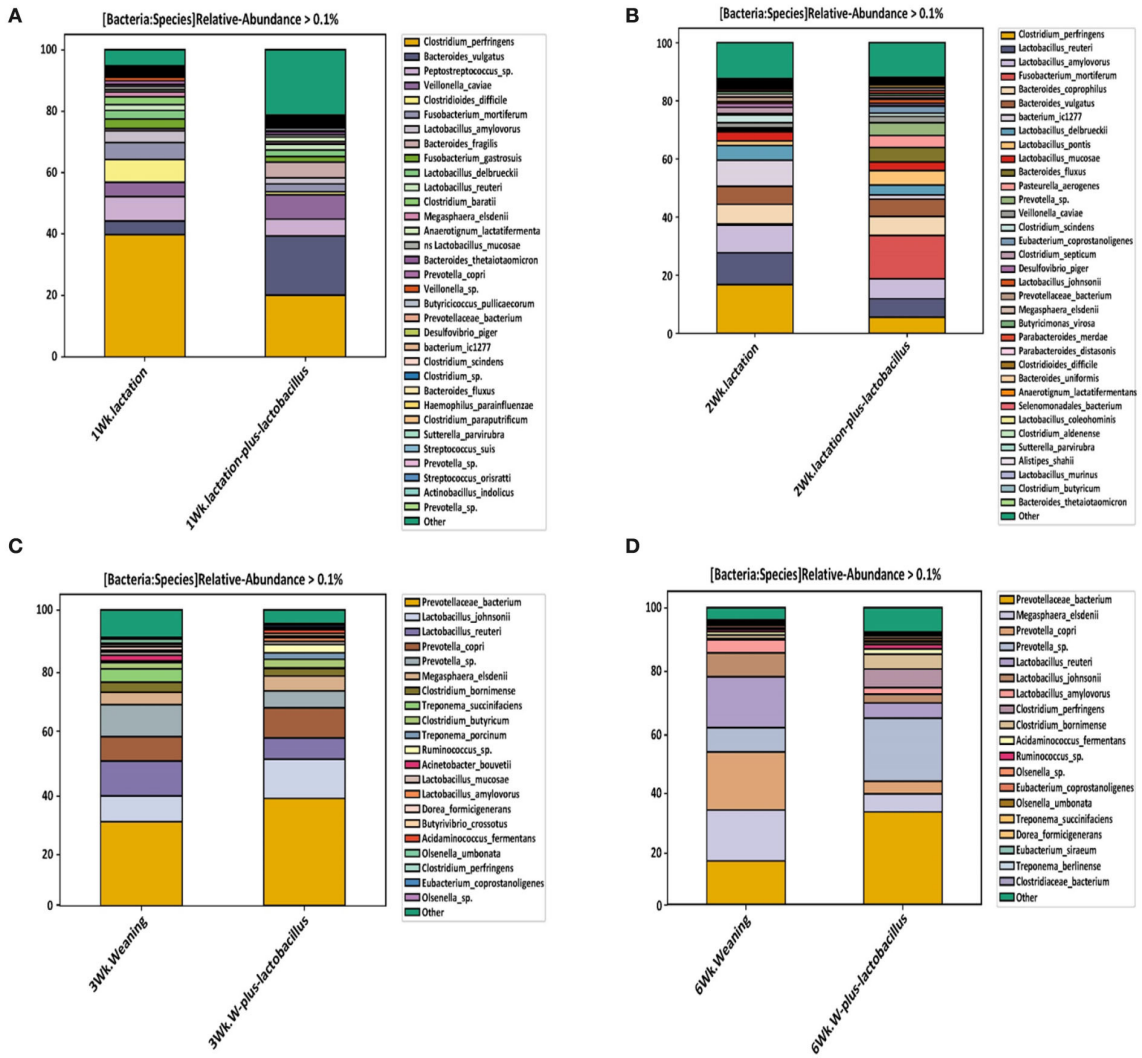
Relative abundance of bacteria at the species level, depending on the duration of probiotic supplement intake [**(A)** week 1, **(B)** week 2, **(C)** week 3, and **(D)** week 6]. These relative abundance bar plots represent the bacterial composition of piglet gut microbiota at the species level. Each legend box at right annotates the bacterial taxonomy in the order of higher bacterial composition.

As shown in Figure 4, we confirmed a difference in the classified bacterial composition at the species level, depending on the type of lactation and weaning diet, similar to the results of the beta-diversity analysis. In the bacterial composition identified during the lactation period (the first and second growth weeks), we focused on changes in the relative frequencies of *Clostridium perfringens* and *Clostridium difficile*. Baker et al. (2013) reported that piglets born to sows and fed a diet supplemented with *Bacillus subtilis* resulted in an increased abundancy of *Lactobacillus* spp. and a reduced abundance of *Clostridium perfringens* in the ileum. In addition, Choudhury et al. (2021) noted that suckling pigs receiving a creep feed modulated the population of *Ruminococcus, Lachnospira, Lachnospiraceae, Roseburia, Papillibacter, Eubacterium*, and *Prevotella* in colonic digesta, which was associated with their intestinal development at weaning. These results indicate that microbiota can be manipulated in early life, inducing long-lasting effects. Moreover, relative frequency reduction rates of *Clostridium perfringens* of 17.8% and 6.3% were observed in the TRT and CON group piglets at the end of each growth week, respectively. The relative frequency of *Clostridium difficile* was, on average, 6.5 and 0.8% in the control and TRT groups, respectively, at the first growth week, showing a difference between the groups. However, *Clostridium difficile* showed no relative intestinal frequency in both the comparison groups in other growth states (Figure 4 and Supplementary Table S3). These bacterial species have a possibility to cause various intestinal diseases such as inflammatory bowel disease (IBD) in host animals (Rood and Cole, 1991). In particular, *Clostridium difficile* is considered a harmful bacterial strain that can induce intestinal dysbiosis in various livestock, including pigs, because intestinal diseases are accompanied by diarrhea (Songer and Anderson, 2006; Alvarez-Perez et al., 2009). In this respect, we confirmed, through these classification results for each taxonomic rank, that a *Lactobacillus* strain-supplemented diet helped to create an intestinal bacterial environment that could positively affect the health promotion of early piglets (during the lactation period) after birth.

## Conclusion

Our results demonstrate that the provision of SM with (1 × 10^9^ CFU/g) *Lactobacillus* sp. supplementation could enhance the body weight and daily gains of neonate piglets at birth and at weaning (week 3), thereby improving their gut health. In addition, *Escherichia-Shigella, Clostridium sensu stricto 1*, and *Bacteroides* were confirmed as the major dominant bacterial genera at the genus level, in both comparison groups at week 1. However, during week 2, the relative proportion of *Escherichia-Shigella, Clostridium sensu stricto 1*, and *Proteobacteria* decreased, whereas those of *Lactobacillus* and *Bacteroidota* increased in piglets receiving the probiotic supplement. Furthermore, *Firmicutes, Proteobacteria*, and *Bacteroidota* phyla were dominant in both comparison groups at week 2, whereas at week 6, *Proteobacteria* showed a slightly increased relative proportion. Moreover, *Prevotella* was confirmed as the major dominant bacterial genus in both groups at weeks 3 and 6. Based on this result, we suggest that nourishing neonates with synthetic milk and (1 × 10^9^ CFU/g) *Lactobacillus* sp. probiotic from birth to 21 days would be beneficial to enhance the gut health of piglets and to overcome post-weaning mortality.

## Data availability statement

All standard sequence formats (. fastq) files generated by Illumina Miseq containing all raw sequence reads have been deposited at the National Center for Biotechnology Information (NCBI) and the data can be accessed through Bio-Project accession number PRJNA901512.

## Ethics statement

The experimental protocol (No: Dk-2-2029) used in this study was approved by the Animal Care and Use Committee of Dankook University, Cheonan, South Korea.

## Author contributions

IK and KH: conceptualization and methodology. JS and JJ: formal analysis. VS and SM: writing—original draft. VS, JS, and SM: investigation. KH and SM: reviewing and editing. All authors reviewed the manuscript. All authors contributed to the article and approved the submitted version.

## Funding

This research was supported by Basic Science Research Capacity Enhancement Project through Korea Basic Science Institute (National research Facilities and Equipment Center) grant funded by the Ministry of Education (Grant No. 2019R1A6C1010033) and the Department of Animal Resource and Science was supported through the Research-Focused Department Promotion and Interdisciplinary Convergence Research Projects as a part of the University Innovation Support Program for Dankook University in 2022.

## Conflict of interest

The authors declare that the research was conducted in the absence of any commercial or financial relationships that could be construed as a potential conflict of interest.

## Publisher's note

All claims expressed in this article are solely those of the authors and do not necessarily represent those of their affiliated organizations, or those of the publisher, the editors and the reviewers. Any product that may be evaluated in this article, or claim that may be made by its manufacturer, is not guaranteed or endorsed by the publisher.
