# Supplementary material for: Nourishing neonatal piglets with synthetic milk and Lactobacillus sp. at birth highly modifies the gut microbial communities at the post-weaning stage
Source: Front Microbiol. 2022 Nov 30;13:1044256. doi: 10.3389/fmicb.2022.1044256 (PMC9748437; doi:10.3389/fmicb.2022.1044256)
Supplement: Supplementary file 4 [file Presentation_1.pdf]

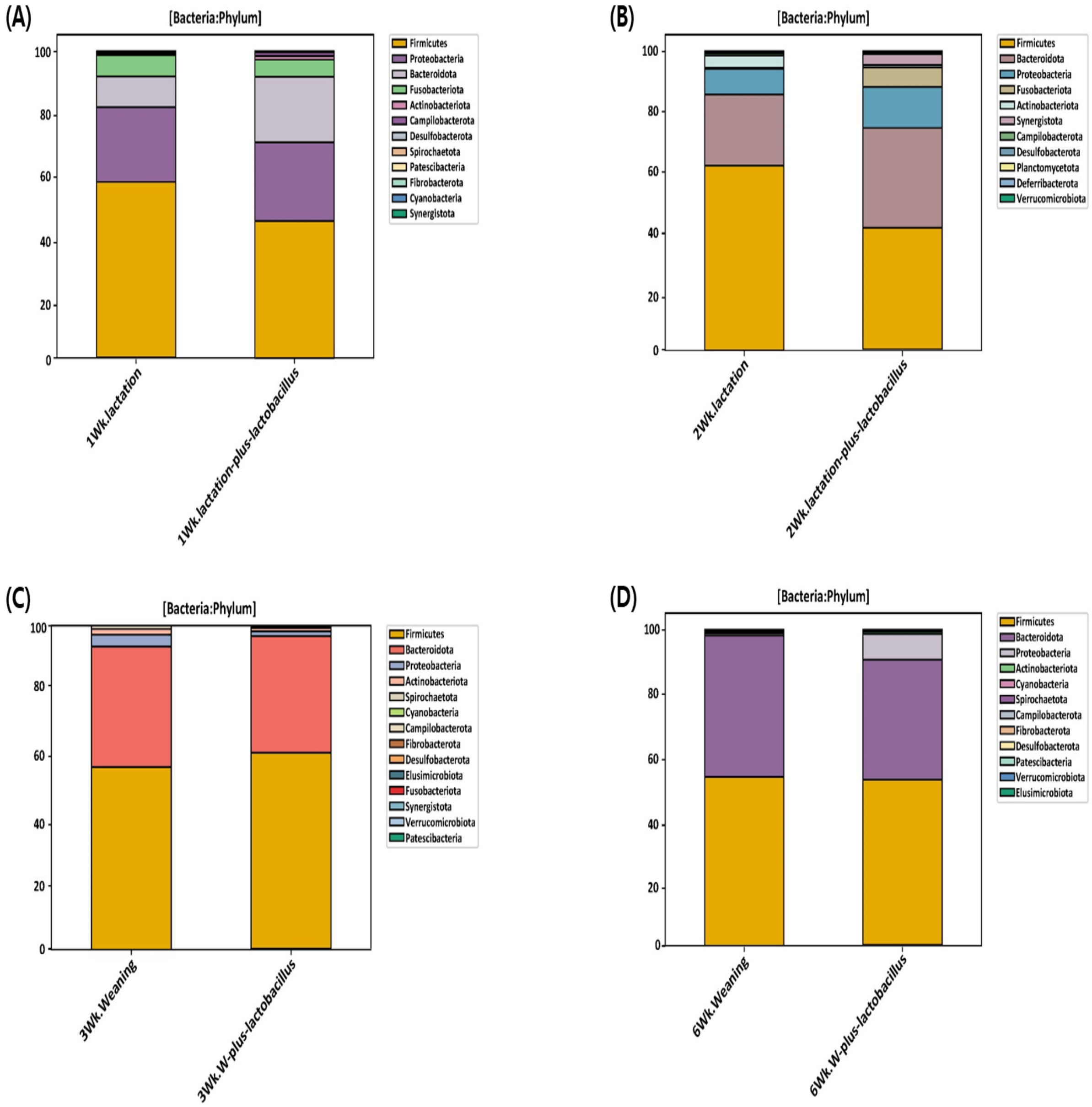

Supplementary Fig. S1. Relative abundance of bacteria at the phylum level, depending on the duration of probiotic supplement intake (A. week 1, B. week 2, C. week 3, and D. week 6). These relative abundance bar plots represent the bacterial composition of piglet gut microbiota at the phylum level. Each legend box displays bacterial taxonomy in the order of bacterial customs classified as a high proportion.

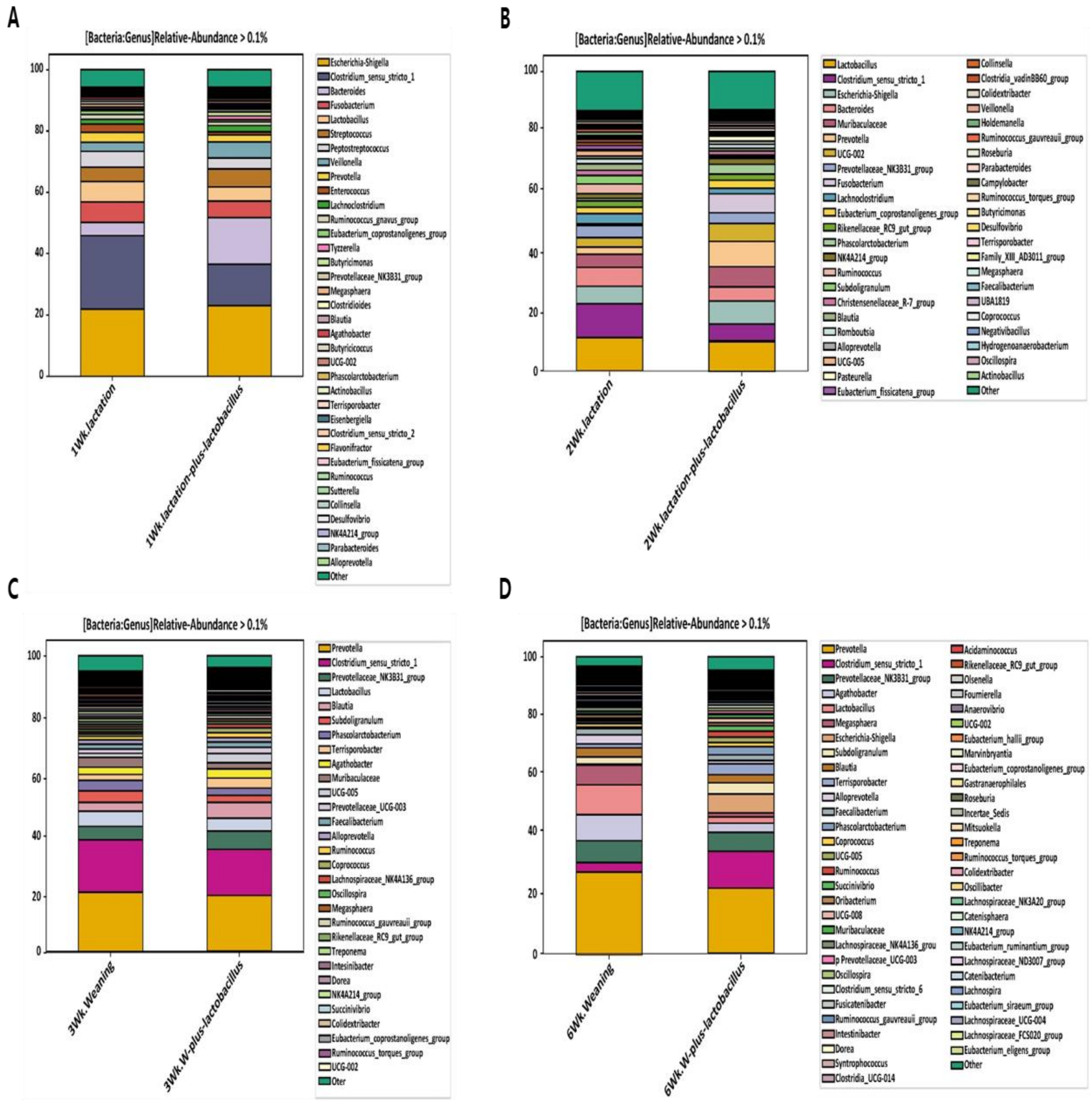

Supplementary Fig. S2. Relative abundance of bacteria at the genus level, depending on the duration of probiotic supplement intake (A. week 1, B. week 2, C. week 3, and D. week 6). These relative abundance bar plots represent the bacterial composition of piglet gut microbiota at the genus level. Each legend box displays bacterial taxonomy in the order of bacterial customs classified as a high proportion
